# Use of *in-vitro* experimental results to model *in-situ* experiments: bio-denitrification under geological disposal conditions

**DOI:** 10.1186/2193-1801-2-339

**Published:** 2013-07-24

**Authors:** Kaoru Masuda, Hiroshi Murakami, Yoshitaka Kurimoto, Osamu Kato, Ko Kato, Akira Honda

**Affiliations:** Kobelco Research Institute Inc, 1-5-5, Takatsuka-dai, Nishi-ku, Kobe, Hyogo, Japan; Kobe Steel, Ltd, 2-2-4, Wakinohama-Kaigandori, Chuo-ku, Kobe, Hyogo, Japan; Nara Institute of Science and Technology, 8916-5, Takayama-cho, Ikoma, Nara, Japan; Japan Atomic Energy Agency, 4-33, muramatsu, Tokai-mura, Naka-gun, Ibaraki, Japan

**Keywords:** Geological disposal, Nitrates, Denitrification, Microorganism, Modeling

## Abstract

Some of the low level radioactive wastes from reprocessing of spent nuclear fuels contain nitrates. Nitrates can be present in the form of soluble salts and can be reduced by various reactions. Among them, reduction by metal compounds and microorganisms seems to be important in the underground repository. Reduction by microorganism is more important in near field area than inside the repository because high pH and extremely high salt concentration would prevent microorganism activities. In the near field, pH is more moderate (pH is around 8) and salt concentration is lower. However, the electron donor may be limited there and it might be the control factor for microorganism's denitrification activities. In this study, *in-vitro* experiments of the nitrate reduction reaction were conducted using model organic materials purported to exist in underground conditions relevant to geological disposal. Two kinds of organic materials were selected. A super plasticizer was selected as being representative of the geological disposal system and humic acid was selected as being representative of pre-existing organic materials in the bedrock. Nitrates were reduced almost to N_2_ gas in the existence of super plasticizer. In the case of humic acids, although nitrates were reduced, the rate was much lower and, in this case, dead organism was used as an electron donor instead of humic acids. A reaction model was developed based on the *in-vitro* experiments and verified by running simulations against data obtained from *in-situ* experiments using actual groundwaters and microorganisms. The simulation showed a good correlation with the experimental data and contributes to the understanding of microbially mediated denitrification in geological disposal systems.

## Introduction

Some of the low level radioactive waste (LLW) from reprocessing of spent nuclear fuel is planned to be disposed of in a purpose built repository at a depth of more than 300 m in the geosphere (geological disposal). In such LLW, nitrates can be present in the form of soluble salts and change to various redox states, such as N(V) as nitrate (NO_3_^–^), N(III) as nitrite (NO_2_^–^), N(0) as nitrogen gas (N_2_), and/or N(−III) as ammonia (NH_3_). It is therefore necessary to understand the fate of NO_3_^–^ and any changes it can provide to the redox environment of the geological disposal system (Japan Atomic Energy Agency and The Federation of Electric Power Companies of Japan 2007). In earlier works of some of the current authors, experimental investigations of the chemical reaction between NO_3_^–^ species and coexisting metallic materials (mainly carbon steel) were used to underpin numerical modeling of the NO_3_^–^ reduction reaction in combination with the corrosion of carbon steel (Honda et al. 2006;Honda et al. 2009;Honda et al. 2011).

The role played by microorganisms in the NO_3_^–^ reduction reaction has been recognized as being an important factor in conditioning the underground chemical environments relevant to geological disposal (Chapelle 2000;Pedersen 2005;Hallbeck & Pedersen 2008;Eydal et al. 2009;Nielsen et al. 2006;Pedersen 2012a;Pedersen 2012b). Previous studies of the underground environment have identified the existence of microorganisms below depths of 300 m, including some NO_3_^–^ reducing microorganisms (Nielsen et al. 2006). In order to establish a proper safety case for the geological disposal system, it is therefore important to construct an evaluation model of NO_3_^–^ behavior including the denitrification process mediated by microorganisms.

The purpose of this study is to adopt a bio-denitrification process to the numerical modeling developed for the environment with metals (Honda et al. 2006;Honda et al. 2009;Honda et al. 2011). In the repository areas, high pH (>12) due to cementitious barriers as well as extremely low water activity due to extremely high nitrate concentrations (about 7 mol/dm^3^) might prevent bioactivities. Therefore, the bio-denitrification model of this study is mainly focused on the near field of the repository. For the near field environment, pH was assumed to be around 8 based on the hypothetical groundwater called "Fresh Reducing High pH" in the report (Japan Atomic Energy Agency and The Federation of Electric Power Companies of Japan 2007).

Mathematical models have been developed for NO_3_^–^ reduction by microorganisms (Henze et al. 2000;Duthy 1993;Clement 1977;Andre et al. 2011;Mcguire et al. 2002). Among such models, the activated sludge model (ASM) is reasonably well-known, focusing on the industrial and practical use of managing the denitrification process of municipal sewage (Henze et al. 2000). In the ASM, like other proposed models, under anaerobic conditions, the NO_3_^–^ reduction reaction is understood as the coupling of an oxidation reaction of organic materials (electron donors) and a reduction reaction of NO_3_^–^ (electron acceptor) instead of gaseous oxygen.

One of the experimental studies on the NO_3_^–^ reduction, in an *in-situ* subsurface environment, was carried out at a site denoted the "MICROBE laboratory" (Pedersen 2005;Hallbeck & Pedersen 2008;Eydal et al. 2009; Nielsen et al.^2006^;Pedersen 2012a;Pedersen 2012b) at the Äspö Hard Rock Laboratory (HRL) in Sweden. The HRL is an underground facility for demonstrating, testing and researching high level radioactive waste disposal concepts. The HRL is situated under the island of Äspö on the Baltic coast of Sweden, approximately 400 km south of Stockholm. The HRL extends to a depth of 460 m in crystalline igneous rock, where the MICROBE laboratory was established at a depth of 450 m (Pedersen 2005).

In the current study, two kinds of organic materials were selected to investigate NO_3_^–^ reduction under geological disposal conditions. A super plasticizer used in the construction of cementitious components was selected as being representative of the geological disposal system and humic acid was selected as being representative of pre-existing organic materials in the bedrock. Firstly, a matrix of *in-vitro* experiments was conducted using these organic materials and typical microorganisms to identify key controlling parameters in the denitrification process. Data obtained from the *in-vitro* experiments were used to develop a NO_3_^–^ reduction reaction model. The NO_3_^–^ reduction reaction model was verified by simulating the data obtained from *in-situ* experiments at the Äspö MICROBE laboratory.

## Methods

### Microorganism

Activated sludge sampled from municipal waste water and *Azoarcus tolulyticus* (*A. tolulyticus*), an underground bacteria identified as having NO_3_^–^ reduction activity (Zhou et al. 1995), were used as representative microorganisms. Activated sludge was selected in addition to *A. tolulyticus* because the activated sludge is a mixture of various kinds of microorganisms, there should be at least one bacterium that can acclimatize to any given environment, in this case, conditions relevant to geological disposal. Activated sludge was used after the removal of contaminants by washing five times with physiological saline solution. *A. tolulyticus*, isolated from a subsurface aquifer, was provided from the American Type Culture Collection (ATCC) through Summit Pharmaceuticals International Corporation (SPI). The strain (ATCC-51758) was cultivated in a liquid medium under aerobic conditions following ATCC's instructions. After repeating the above procedure five times, solutions for the experiments were prepared. Turbidity (absorbance at 610 nm) was adjusted to the same value as that of 1000 mg solution of the activated sludge. The concentration of the so obtained solution of *A. tolulyticus* was defined as 1000 mg/dm^3^. For cultivation, typical media used for the evaluation of denitrification activity was used, replacing ammonium ion by sodium ion in order to avoid uncertainty in material balance analysis of nitrogen.

Variation of microorganism concentration was evaluated by Lumitester of Kikkoman Corporation, by which microorganism concentration is evaluated as intensity of luminescence released when the ATP (adenosine triphosphate) is transformed to AMP (adenosine monophosphate) in the presence of luciferaze. In this study, the intensity of luminescence of the initial solution was set to 1.0 and the relative intensity of the sample was evaluated as the variation of microbial population (relative growth rate).

### Samples and reagents

Two kinds of humic acid were provided by the Japanese Humic Substances Society (JHSS) (Watanabe et al. 1944). Humic acids from Dando and Inogashira provided by JHSS were mixed at a mass ratio 1:1. The other humic acid was a reagent grade sodium salt from Aldrich and used as received.

A super plasticizer used for the *in-vitro* experiments was Mity 3000S from Kao Corporation, consisting of polyether compounds containing carboxylic groups as main components. The concentration of total organic carbon (TOC) of the solution was adjusted to a designated value by dilution. In order to respond to a recent view that the molecular weight distribution of organic compounds originated from super plasticizers in the solution of cement pores tends to decrease in comparison with that of the original plasticizers (Suguro et al. 2011;Fujita et al. 2008), the super plasticizer solution for the *in-situ* experiments was prepared by the following procedure. A super plasticizer, Reobuild SF8LS from BASF, was mixed with a Portland cement to adsorb to the cement materials. The cement slurry was made up by mixing the Portland cement and distilled water (mass ratio of cement/water was 2), followed by the removal of soluble alkaline materials (principally Na and K) by replacing the supernatant with fresh distilled water after overnight storage of the slurry. After replacing the supernatant three times, the soluble alkaline component was removed and a cement hydrate containing 50 wt % water was obtained. This cement hydrate was mixed with solution containing 2 wt % of the super plasticizer and 0.2 wt % of ethylene glycol. The mass ratio of the cement hydrate to the solution containing organic materials was 100 to 20. After this mixture was stored for 7 days under ambient conditions, the supernatant was separated by centrifugation to provide the sample solution containing the extracts of the super plasticizer. The molecular weight of organic materials in the solution was 200 to 300, which was similar to that of organics in the solution of cement pores reported previously (Suguro et al. 2011;Fujita et al. 2008). The solution was then bubbled with carbon dioxide to fix the pH to 8 with the precipitate of calcium carbonate removed by filtration using 0.45 μm membrane filter. The final concentration of the solution was set to a designated concentration by removing water through evaporation.

A NO_3_^–^ solution was prepared by dissolution of NaNO_3_ into pure water and pH was adjusted to 8 (except Run1 and Run2, pH7) by NaOH. NaNO_3_ and NaOH used were JIS special grade chemicals from Wako Chemicals Corporation.

### *In-vitro* experiments

In vitro experiments were carried out using ampule experiments and vial experiments described below. To ensure long-term anaerobic conditions and to analyze nitrogen contents, while avoiding air contamination, the so-called ampule experiment (Honda et al. 1999) was conducted (details shown in Table 1). A test solution was made up by mixing of solution containing microorganism and solution containing the nitrate and organics in a glove box (Argon [Ar] atmosphere, oxygen concentration < 0.1 ppm) followed by Ar gas sparging to remove dissolved oxygen from the solution. The solution was then placed in the designated glass tube (volume ≈ 0.08 dm^3^) and the spout fusion sealed to prevent any atmospheric contamination. The sealed glass tubes were then taken out of the glove box and stored in a thermostat oven at 35°C for a designated time of 1–50 days. After storage, the ampule seal was broken in a vacuum chamber connected to the injection port of a gas chromatograph (GC), so that the composition of the production gas could be analyzed without air contamination. For tests to investigate time dependence, ampules were prepared for every experiment run. Shortly after the opening the ampules, NO_3_^–^ and NO_2_^—^ of the solution were analyzed by ion chromatography, NH_3_ by UV spectrometer and TOC by furnace-infrared method. The solid fraction was separated by filtration using a 0.2 μm membrane filter and its C, H and N content was analyzed by a CHN coder (Yanako MT700 CHN).

**Table 1:** **Experimental condition of*in-vitro*tests**

Series	Run	Method	Initial Condition^*1^		Microorganism		Electron Donor				Duration
			pH	Conc. of NO_3_^–^	Activated Sludge	***Azoarcus Tolulyticus***	Glucose	Super Plasticizer	Mixture of HA^*2^	Sodium Salt of HA^*3^	
				(mmol/dm^3^)	(mg/dm^3^)	(mg/dm^3^)	(mg/dm^3^)	(mg/dm^3^)	(mg/dm^3^)	(mg/dm^3^)	(d)
A	1	Ampule	7	20	1000	-	-	0		-	50
	2	Ampule	7	20	1000	-	-	3000		-	6–50
B	3	Ampule	8	10	1000	-	-	-	100	-	10
	4	Ampule	8	50	1000	-	-	-	100	-	10
	5	Vial	8	10	1000	-	-	-	1	-	0–30
	6	Vial	8	10	1000	-	-	-	10	-	0–30
	7	Vial	8	10	1000	-	-	-	100	-	0–30
C	8	Ampule	8	50	-	1000	-	-	-	0	11
	9	Ampule	8	50	-	1000	-	-	-	10	11
	10	Ampule	8	50	-	1000	-	-	-	100	11
	11	Ampule	8	50	-	1000	-	-	-	1000	11
D	12	Vial	8	50	-	1000	100	-	-	-	0.2–7
	13	Vial	8	50	-	1000	-	-	-	100	0.2–7
E	14	Vial	8	50	10	-	-	-	-	0	1–50
	15	Vial	8	50	10	-	-	-	-	10	1–50

^*1^ In every experiments, temperature was controlled at 35 degree-Celcius and sealed gas was Argon. ^*2^ Humic acids (HA) from Japan Humic Substance Association, ^*3^ Reagent grade humic acids from Aldrich.

In addition to the ampule tests, vial tests were also conducted to investigate the effects of using different microorganisms and organic materials (details shown in Table 1). In the vial tests, gases in the liquid and gaseous phase in the vial tubes were replaced by Ar and spouts were sealed with silicon rubber to keep the interior of the tubes anaerobic. The volume of the vial tubes was 0.05 dm^3^ and solution volume was 0.03 dm^3^. After storage for a designated period of 1–50 days at room temperature, the gaseous phase was transferred to a gas bag through a syringe and N_2_ analyzed by GC. Analysis of the liquid phase used the same methods as the ampule tests described above.

As shown in Table 1, ampule and vial tests were carried out in the combination of variation of organic materials and microorganisms in series A to E.

### *In-situ* experiments

A series of *in-situ* experiments were conducted at the MICROBE Laboratory situated at the Äspö HRL (Pedersen 2005). The flow system setup in the MICROBE Laboratory consists of four flow cells; details can be found in Pederson (Pedersen 2005). Flow cells were dried after sterilize with 70% ethanol. Each cell was subsequently filled with crushed (2–4 mm) and heat sterilized (433K for 5 hrs) rocks from the drill core of the test site, to give a total crushed rock mass = 440 g. Groundwater was then fed into the flow cell. The ground water was introduced from drill core into the inlet of the flow cells, drawn from outlet and circulated to drill core. This circulation was carried out for 30 days at a flow rate of approximately 20 ml/min, a pressure of 2.5 MPa and a temperature of 17°C until bio-films had grown on the crushed rock surface. After the one-through circulation was shutoff, the groundwater was circulated in the flow cells. The solution containing NO_3_^–^ and organic materials was injected. The organic materials were extracts from the mixture of super plasticizer and cementitious materials; described in sample and reagents. The solution was sampled periodically and NO_3_^—^ concentration and number of microorganism was analyzed. Sampling periods were 0, 0.9, 3.9, 8.9, 17 and 31 day. Solution sampled through 0.2 μm sterilized filters was used for analyses of chemical species as a microorganism free solution. Total number of cells of microorganisms (TNC) was determined using the acridine orange direct count method (Pedersen & Ekendahl 1990). The quantitative polymerase reactions (Q-PCR) were run in duplicate. Primers for the gene NarG (Henry et al. 2004) were used. The PCR mixture contained 1.0 μL of the primer (10 pmol/μL), 16 ng of DNA, 12.5 μL Stratagene Brilliant SYBR II Q-PCR Mastermix 2X (AH Diagnostics AB, Skarholmen, Sweden), and sterile water to a final reaction volume of 25 μL. Amplification was carried out on a Stratagene Mx35005P Q-PCR thermal cycler (AH Diagnostics AB). The primers were temperature optimized and the products with the standard samples were checked on agarose gels to verify the size of the fragments. The dissociation curves (melting curves) were also checked to evaluate the specificity of the primers. *Pseudomonas* fluorescence was used as standard.

## Results

### *In-vitro* experiments

Analytical data obtained from the *in-vitro* experiments are shown in Table 2 (ampule tests) and Table 3 (vial tests). The results from each series are described separately below.

**Table 2:** **Analytical results of*in-vitro*tests (glass ampule tests)**

Series	Run	Time (d)	Liquid phase						Gas phase			Solid phase			Relative growth rate of microorganism^*1^
			pH	TOC	T-N	NO_3_^–^	NO_2_^–^	NH_3_	N_2_	CO_2_	NH_3_	C	H	N	
				mmol/dm^3^					mmol/dm^3^			mmol/dm^3^			
A	1	50	7.7	3.2	22	6.2	2.0	19	5.4	ND	ND	18	44	3.8	0.67
	2	6	6.9	130	13	8.6	0.7	3.4	3.4	ND	ND	24	44	3.2	0.63
	2	28	7.2	140	2.0	ND	ND	3.4	9.0	ND	ND	20	38	2.4	0.3
	2	50	7.3	140	2.4	ND	ND	3.4	8.4	ND	ND	24	44	3.0	0.44
B	3	10	8.7	2.0	9.4	3.0	0.2	2.8	3.6	ND	ND	17	38	5.2	0.32
	4	10	8.5	2.0	50	44	1.6	2.6	2.8	ND	ND	18	36	2.0	0.27
C	8	1	8.3	2.2	47	48	0.4	0.1	-	-	-	2.6	5.2	0.6	0.53
	8	11	8.3	3.0	47	44	2.2	0.7	0.4	ND	ND	0.9	1.4	0.2	0.04
	9	1	8.3	1.7	47	46	0.4	0.1	-	-	-	1.6	2.8	0.2	0.85
	9	11	8.4	3.2	47	44	2.2	0.7	0.4	ND	ND	0.6	0.5	0.2	0.055
	10	1	8.4	5.0	49	48	0.2	0.1	-	-	-	1.3	2.0	0.2	0.74
	10	11	8.0	7.6	47	42	4.0	1.1	0.5	ND	ND	2.2	2.4	0.6	0.058
	11	1	8.3	30	50	50	ND	0.2	-	-	-	1.0	1.7	0.1	0.63
	11	11	8.5	34	49	46	0.4	1.1	0.5	ND	ND	3.2	5.0	0.3	0.013

*1 Population of microorganisms relative to the initial solution.

**Table 3:** **Analytical results of vial tests**

Series	Run	Time (d)	0	0.2	1	3	7	10	14	20	22	30	50
		Item											
B	5	pH	8.0			7.8	7.8	7.9		8.0		8.0	
		NO_3_^–^ (mmol/dm^3^)	0.50	0		0.35	0.25	0.25		0.20		0.15	
		Relative growth rate ^*1^	1.0			2.5	0.5	0.9		0.3		0.3	
	6	pH	8.0			7.7	7.8	7.9		7.9		8.1	
		NO_3_^–^ (mmol/dm^3^)	0.50			0.35	0.30	0.30		0.20		0.15	
		Relative growth rate	1.0			1.8	0.5	0.8		0.3		0.2	
	7	pH	8.0			7.7	7.8	7.8		7.9		8.0	
		NO_3_^–^ (mmol/dm^3^)	0.55			0.35	0.30	0.25		0.20		0.10	
		Relative growth rate	1.0			1.1	0.3	0.4		0.2		0.2	
D	12	pH	8.0	7.0	8.0	8.3	8.8						
		NO_3_^–^ (mmol/dm^3^)	50	43	3	2	2						
		Relative growth rate	1.0	3.10	3.30	0.10	0.30						
	13	pH	8.0	7.9	8.4	8.7	8.9						
		NO_3_^–^ (mmol/dm^3^)	50	43	45	43	38						
		Growth rate	1.0	0.59	0.87	1.90	0.16						
E	14	pH	8.0		9.2	8.9	8.7	8.6	8.6		8.3	8.7	8.6
		NO_3_^–^ (mmol/dm^3^)	10		10.4	10.1	9.54	10.5	10.4		10.4	10.1	10.4
		Relative growth rate	1.00		1.20	0.61	0.43	0.28	0.28		0.17	0.09	0.07
		N_2_ (mmol/dm^3^)											0.14
	15	pH	8.0		9.1	9.0	8.8	8.8	8.7		8.7	8.8	8.8
		NO_3_^–^ (mmol/dm^3^)	10		10.3	10.6	10.3	10.6	10.5		10.4	10.3	10.7
		Relative growth rate	1.00		1.26	0.71	0.46	0.34	0.27		0.18	0.11	0.08
		N_2_ (mmol/dm^3^)											0.24

*1 Population of microorganisms relative to the initial solution.

**Figure 1:**
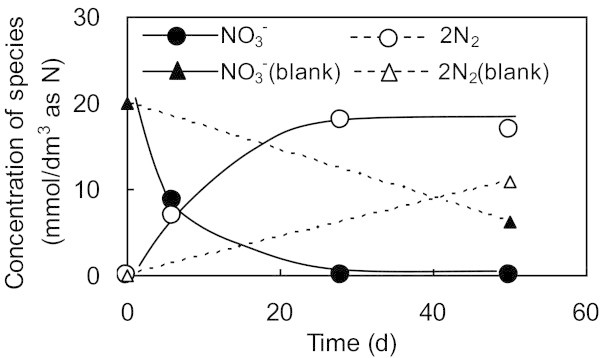
**Comparison of nitrate utilization and nitrogen gas evolved with and without super plasticizer as an electron donor in 1.0 mmol/dm**^**3**^**of nitrate solutions and 1000 mg/dm**^**3**^**of activated sludge as the microorganism.** Run 2 was carried out with the electron donor and Run1 was carried out without the electron donor (blank). 2N_2_ is used for amount of N_2_ evolved in order to directly compare the mass balance with NO_3_^-^.Lines are shown to help identify the general trends shown by the data.

**Figure 2:**
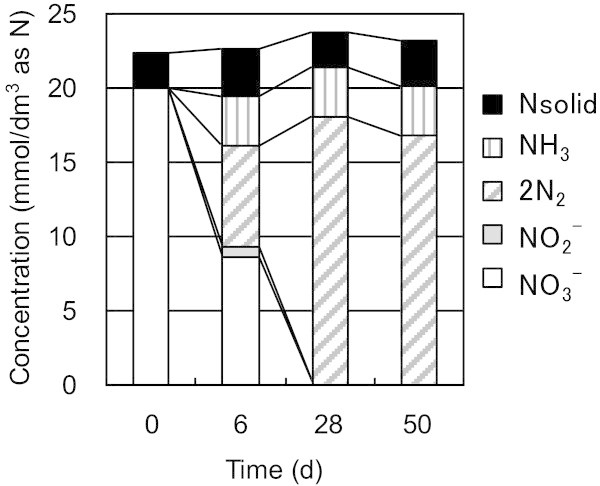
**Material balance of nitrogen species during Run 2 in the presence of super plasticizer as an electron donor, where the initial concentrations of nitrate and micro organism were 20 mmol/dm**^**3**^**and 1000 mg/dm**^**3**^**, respectively.**

In series A, Run 1 was run as a blank and Run 2 contained super plasticizer in order to elucidate the potential of super plasticizer as an electron donor on the behavior of NO_3_^–^ reduction using activated sludge as the microorganism. The temporal changes in the composition of nitrogen compounds in Run 1 and Run 2 are shown in Figure 1. Although NO_3_^–^ was consumed in the blank test (Run 1), more NO_3_^–^ was reduced to produce more N_2_ by the presence of super plasticizers (Run 2). The consumption of NO_3_^–^ in the blank test will be discussed in more detail later. The material balance of nitrogen classified according to species (NO_3_^–^, NO_2_^–^, NH_3_, N_2_ or solid phase [Nsolid]) is shown in Figure 2. Nsolid was calculated from nitrogen contents of the solid fraction. In the Figure 2, 2N_2_ was used for direct comparison in atomic nitrogen base. In Run 2, NO_3_^–^ initially accounted for all nitrogen not in the solid phase. The production of NH_3_ and the appearance of NO_2_^–^ was limited to the first 6 days of reaction. Consequently, the NO_3_^–^ decrease could be broadly correlated with the production of N_2_.

**Figure 3:**
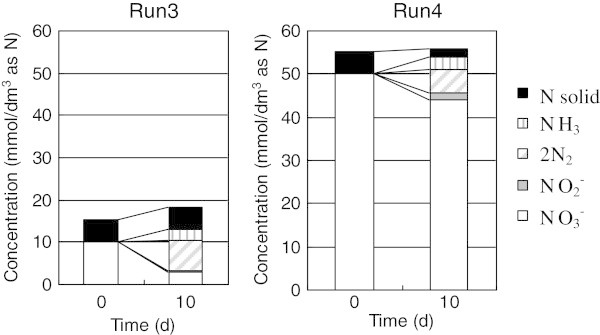
**Material balance of nitrogen species during glass ampoule experiments (Run 3 and 4) in the presence of humic acid.** Run3: Initial concentrations of NO_3_^–^, activated sludge and humic acid were 20 mmol/dm^3^, 1000 mg/dm^3^ and 100 mg/dm^3^ as carbon, respectively. Run4: Initial conditions = Run3, except for NO_3_^–^ = 50 mmol/dm^3^.

**Figure 4:**
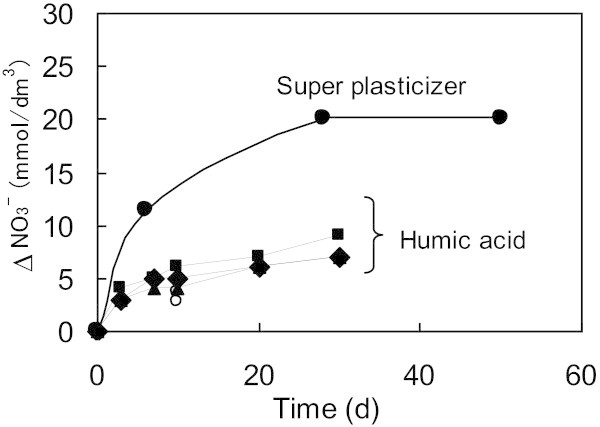
**Comparison of humic acid and super plasticizer as an electron donor from the viewpoint of nitrate utilization with activated sludge as microorganism.** Run 2 (shown as black circles and solid line) was carried out with super plasticizer and Run 3–7 (shown as dotted lines) were carried out with humic acids under the conditions shown in the Table 1. Lines are shown to help identify the general trends shown by the data.

In series B (Runs 3–7), humic acids were used as electron donors with activated sludge as the microorganism. Ampule tests were carried out in Run 3 and Run 4 with changes in the initial NO_3_^–^ concentration, while vial tests were carried out in Run 5–7 to investigate temporal changes. The nitrogen balance in Run 3 and 4 (Figure 3) shows that the main reaction is the reduction of NO_3_^–^ to N_2_ in the case of the humic acids; as was found for the super plasticizers (Figure 2). Comparison of the NO_3_^–^ reduction rate in the presence of humic acid with super plasticizers (Figure 4) demonstrates that the amount of NO_3_^–^ reduced in any of Runs 5–7 was much lower than that of Run 2. Furthermore, there was no dependence of NO_3_^–^ reduction rate on the initial NO_3_^–^ concentration (Figure 4).

**Figure 5:**
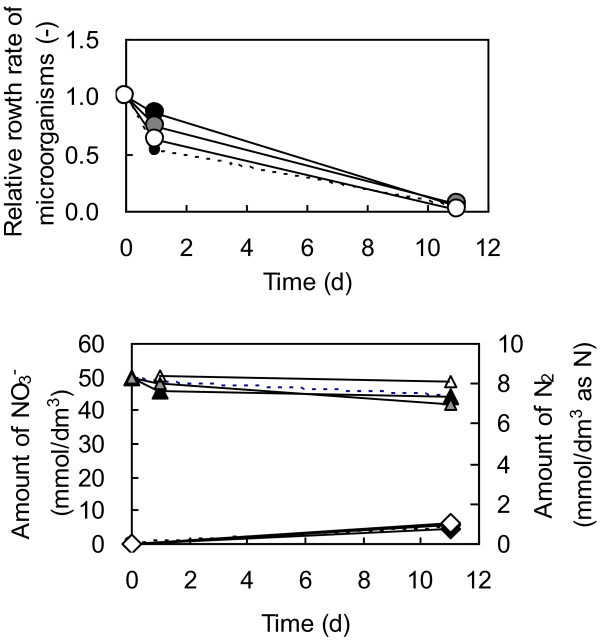
**Variation of nitrogen species and microorganism in the presence of humic acid as an electron donor (Run8-11).** Initial humic acid concentrations are 0 (small black circles and dotted line), 10 (black marks), 100 (gray marks) and 1000 (white marks) mg/dm^3^. Other initial conditions are the same: initial concentrations of NO_3_^–^ and microorganism (*Azoarcus tolulyticus*) were 50 mmol/dm^3^and 1000 mg/dm^3^, respectively. Lines are shown to help identify the general trends shown by the data.

**Figure 6:**
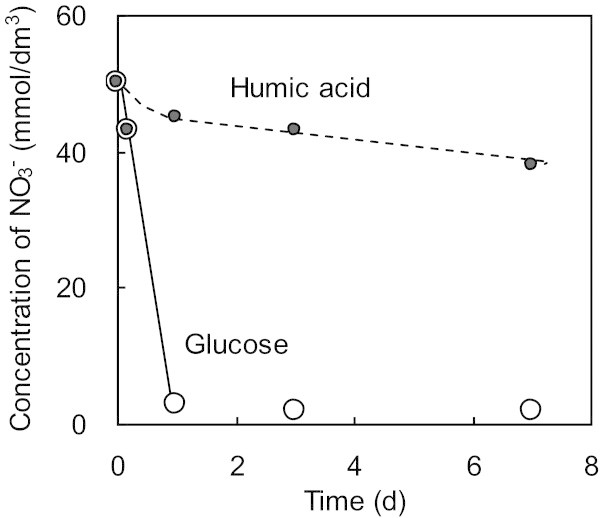
**Comparison of nitrate utilization between glucose (Run 12) and humic acids (Run 13) as electron donors with*Azoarcus tolulyticus*as the microorganism.** Initial nitrate concentrations of nitrate, electron donors and microorganism are 50 mmol/dm^3^, 100 mg/dm^3^ and 1000 mg/dm^3^, respectively, by vial experiments. Lines are shown to help identify the general trends shown by the data.

In series C (Runs 8–11), tests were carried out using humic acids with *A. tolulyticus* as the microorganism. Run 8 was a blank test without humic acids and Runs 9–11 were with different concentrations of humic acids. There was no significant difference in the NO_3_^–^ reduction reaction and the growth of microorganisms between the blank (Run 8) and those with humic acids (Runs 9–11) (Figure 5). In each case, the concentration of microorganisms after eleven days decreased below one tenth of the initial value. By contrast, the activity of *A. tolulyticus* was confirmed in the experiments in series D (Runs 12 and 13) (Figure 6). In this series, glucose was used in Run 12 because it is easily decomposable in comparison with the humic acid used in Run 13. As shown in Figure 6, NO_3_^–^ was reduced within two days in the presence of glucose, significantly faster than in the presence of humic acids. This result indicates that *A. tolulyticus* can adequately reduce NO_3_^–^ in the presence of easily decomposable organics. It was therefore concluded that no significant denitrification using humic acids could be observed and might be due to a lack of decomposable organics initially introduced. This lack of initial decomposable organics could be a consequence of a dissolved portion of dead microorganisms acting as electron donors instead of the humic acids.

**Figure 7:**
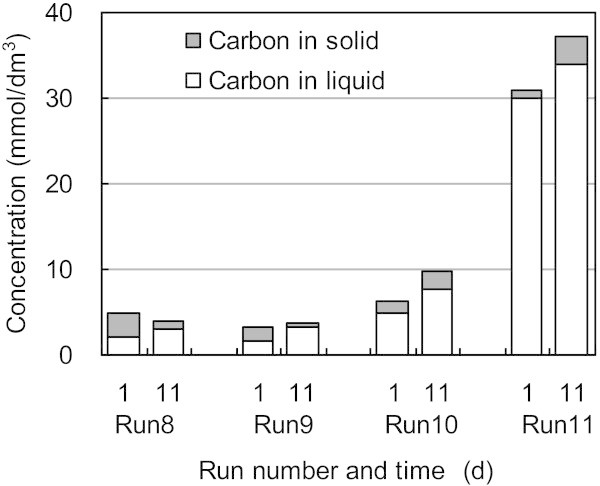
**Change in distribution of organic carbon in experiments using the humic acid as electron donors, where the concentrations of the humic acid were 0 (Run 8), 10 (Run9), 100 (Run10) and 1000 (Run11) mg/dm**^**3**^**.**

Figure 7 shows the material balance of organic carbon in the presence of humic acid. Humic acids correspond to the soluble carbon component, because humic acids used for these experiments were sodium salts from Aldrich, and the microorganisms correspond to the solid carbon. Based on these assumptions, it is shown that carbon in the solid decreased and that carbon in the solution increased with time, indicating that some portion of the solid carbon dissolved. These results imply that soluble carbon produced from the debris of dead microorganisms is consumed by living microorganisms with NO_3_^–^ reduction occurring to some extent.

In order to evaluate a consumption rate, the initial concentration of microorganisms should be low enough to avoid influence of the reuse of soluble organics from dead microorganisms. Thus, in additional set of experiments, series E (Runs 14 and 15), the initial concentration of microorganisms was set to 10 mg/dm^3^.

**Figure 8:**
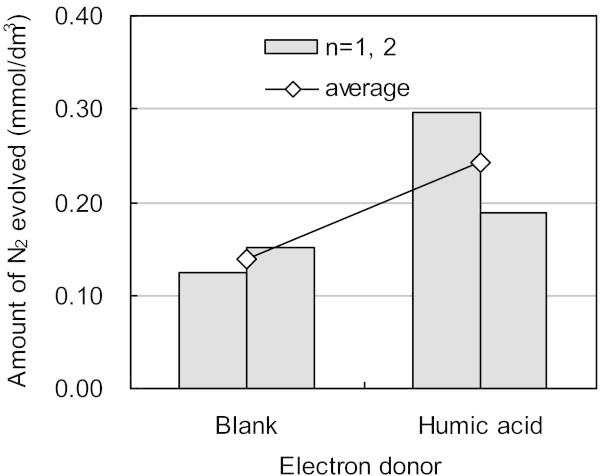
**Comparison of evolved nitrogen gas between blank (Run 14) and humic acid (Run 15).** Two analytical data under each condition are shown in the bar graphs and their averages plotted by.

In Run14 and 15, vial tests were carried out for 50 days to compare NO_3_^–^ reduction rate without and with the addition of humic acids, respectively (Figure 8). The amount of N_2_ gas produced was more in Run 15 than in Run 14 and suggests that the consumption rate of humic acids by microorganisms as electron donors might not be zero, but slower than that of other organic materials including organics from dead microorganisms.

### *In-situ* experiments

Results of the NO_3_^–^ reduction reaction using microorganisms from actual groundwater at Äspö HRL and an extract of the mixture of super plasticizers and cementitious materials are shown in Tables 4 and 5. Table 4 shows concentrations of NO_3_^–^, NO_2_^–^ and NH_3_ and Table 5 shows data obtained from TNC and Q-PCR analyses. Samples were named S-1 to S-6 with regard to sampling times from 0 to 30 days (Table 5). Sample S-1 is the solution sampled before the injection of NO_3_^–^ and super plasticizer solution. After the injection of NO_3_^–^ and organics, NO_3_^–^ concentration decreased and the number of microorganisms increased and so confirmed that the extract of super plasticizer acts as an electron donor for the NO_3_^–^ reducing bacteria from actual groundwater.

**Table 4:** **Concentrations of nitrogen compounds from*in-situ*experiments**

Sample No.	Sampling time (d)	NO_3_^–^	NO_2_^–^	NH_3_
		mmol/dm^3^	mmol/dm^3^	mmol/dm^3^
S-1	0	0.000013	0.000012	0.00200
S-2	0.9	9.05	0.0056	0.00078
S-3	3.9	9.05	0.0051	0.00027
S-4	8.9	8.79	0.0043	0.00028
S-5	17	7.95	0.0016	0.00018
S-6	31	7.69	0.0044	0.00048

**Table 5:** **Analytical result of TNC and Q-PCR**

Sample No.	Time (d)	TNC in solution		Q-PCR of NarG-DNA			
				In solution		On biofilm	
		cells/cm^3^	σ (n=3)	copies/g	σ(n=4)	copies/g	σ(n=8 or 16)
S-1	0	130	32	3.94E05	2.27E05	2.26E08	1.85E08
S-2	0.9	190	54	3.63E05	1.17E05	1.95E08	7.70E07
S-3	3.9	1,400	99	7.98E05	3.12E05	3.93E08	3.53E08
S-4	8.9	2,900	140	8.93E05	7.41E05	4.27E08	3.22E08
S-5	17	4,000	1,300	3.37E06	2.19E06	6.32E08	2.72E08
S-6	31	5,100	710	4.31E07	2.84E07	1.35E09	7.55E08

## Discussion

### Reaction model outline

The reaction was modeled with reference to the anaerobic respiration process in ASM1 and ASM3C [12.13]. The ASM1 is the most fundamental model and ASM3C is constracuted with TOC-base (unit of both organics and microorganism is in TOC (g-C/dm^3^ or mol-C/dm^3^). Under anaerobic conditions where the electron acceptor is limited to NO_3_^–^, the growth rate of microorganisms with time, dxB/dt(mol-TOC/dm^3^/d), without considering their death, is modeled as (Henze et al. 2000;Duthy 1993):1

where k3 is a rate constant (d^-1^), sS, sNO3, xB are concentrations of soluble organic materials (mol-TOC/dm^3^), NO_3_^–^ (mol-N/dm^3^) and microorganism (mol-C/dm^3^), respectively, and KsS and KsNO3 are half saturation concentration of organic materials (mol-TOC/dm^3^) and NO_3_^–^ (mol-N/dm^3^), respectively. Describing the death rate of microorganisms by bH·xB, where bH is the death rate constant (d^-1^), Equation (1) can be rewritten as:2

Soluble organics, sS, are consumed by the activity of bacteria and their rate of change is described by:3

where *x* is the specific growth rate given as the ratio of amount of organics to be transformed to the amount of consumed organics (mol/mol).

Furthermore, the changing rate of NO_3_^–^ (electron acceptor) concentration and those of soluble organics (electron donor) can be related by4

where *z* [(mol-N)/(mol-C)] is the N/C ratio of the above pair of reactions.

According to experimental studies of this work, when the above reactions are modeled, the following items should be considered:

1. The growth rate and the NO_3_^–^ reduction rate depend on the consumption of organic materials. Among the organic materials examined in this study, humic acids were not easily consumed.
2. By contrast, when no organic material exists or only hard-bio-decomposable organic materials exist, like humic acids, soluble organics produced from dead microorganisms can act as electron donors. In view of the results obtained in this study, organic materials can be listed in the order of ease of microorganism usage as:

glucose > plasticizer > organic fraction produced from dead microorganisms > humic acids

3) Biogenic reduction products of NO_3_^–^ are almost exclusively N_2_, but in some cases, depending on the species of microorganisms for example, products may include small amounts of NO_2_^–^ or NH_3_.

In order to construct a reaction model considering these phenomena, the standard model, as adopted in the ASM, needs to be modified by the addition of reaction processes and parameters as described below.

1. Organics should be categorized based on the degradability and reaction rate equation and parameters should be assigned to each category. From the viewpoint of geological disposal, important organics are super plasticizers, humic acids and also organics originating from dead microorganisms.
2. In the ASM, production process of soluble organic materials from dead cell is described as hydration reaction of solid organics of dead cell. However, the results of this study suggest that it is also necessary to consider the direct production process of soluble organics from dead microorganisms. It is understood that this direct process may be important in the case of geological disposal environments where the existence of easy-biodegradable organic materials may be low.
3. The NO_3_^–^ reduction reaction by microorganisms is known to follow the sequence of NO_3_^–^ → NO_2_^–^ → N_2_. In general, however, bio-modeling, including the ASM approach, adopts the direct process of N_2_ production from NO_3_^–^ reduction, neglecting any intermediate steps. Similarly, the modeling in this study adopts the direct process, given that NO_2_^–^ production was very low, even when actual groundwater was used (shown in Table 4).

### Model parameters

Before constructing a predictive model, data from the *in-situ* experiments were adapted for use in accordance with the following procedure:

1. The groundwater flow experiment was conducted for thirty days, after which time bio-films had grown on the crushed rock surface. Therefore, the concentration of microorganisms was taken as the sum of those in the solution and in the bio-films.
2. Concentration of microorganisms in the bio-films on TNC (total number of cells of microorganisms described in "*in-situ* experiments") base was calculated from the Q-PCR of solution and bio-films correlating with TNC of the solution. Bacteria concentrations from the sum of solution and bio-films on the TNC base were estimated from the sum of Q-PCR data of solution and bio-film, applying a factor obtained by correlating TNC and Q-PCR of the same solution.
3. TNC was transformed to TOC because the model of this study uses microorganism concentrations in TOC base. Assuming that half of the decrease of TOC in the solution was used for the growth of bacteria (x = 0.5), a correlation factor was obtained by calculating the ratio of TOC decrease and microorganism increase throughout the experiment. The microorganism concentration of each time step was then transformed to TOC base using the above correlation factor.
4. A ratio of NO_3_^–^ required to that of carbon used for bacteria growth was also calculated from the experimental data.

The resulting adapted data used for the predictive model are shown in Table 6.

**Table 6:** **Preparation of data analysis**

Sample	Time (d)	TOC (gC/dm^3^)	Count of microbe as TNC (cells/dm^3^)		Count of microbe as TOC	NO_3_^-^(g-N/dm^3^)
			In solution (TNC method)	In total (correlated to TNC)	In total (gTOC/dm^3^)	
S-1	0.0	0.0004	1.30E+08	2.42E+10	-	-
S-2	0.9	0.190	1.84E+08	2.09E+10	1.22E-03	0.125
S-3	3.9	0.194	1.15E+09	4.21E+10	2.46E-03	0.125
S-4	8.9	0.191	2.20E+09	4.58E+10	2.67E-03	0.122
S-5	18.0	0.185	2.86E+09	6.91E+10	4.03E-03	0.115
S-6	31.0	0.180	3.41E+09	1.68E+11	9.82E-03	0.113
	increase of amount	−0.014		1.22E+11	8.60E-03	−0.012
	Parameters^*,†^	(1)		(2)		(3)

*In-situ* test using extract of cement agent.

^*^TOC/Microbe correlation factor = −0.5×(1)÷(2) = 5.74E-14.

^†^N/C = (3)÷(1) = 0.86≒1.0 (g-N)/(g-C).

### Model verification

**Figure 9:**
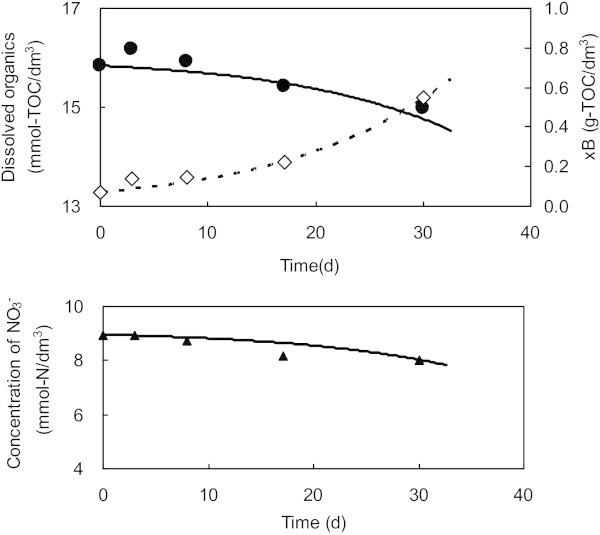
**Comparison between experiments and simulation results.** Data from the in-situ experiment using super plasticizer as an electron donor are plotted as symbols, while simulation results are shown as lines. Representative parameters to be used in Equation (3) are: k3 = 0.1 d^-1^, bH = 0.005 d^-1^ and N/C = 1 gC/gN.

The reaction model was applied to the interpretation of *in-situ* experimental results. From an estimated N/C = 1.0 (Table 6) and using k3 = 0.1 d^-1^ and bH = 0.005 d^-1^, parameters concerned with the growth rate of microorganisms, provided a good correlation with the prepared *in-situ* data (Figure 9). As indicated by the experiments, microorganisms grew very slowly using the extract of the mixture of super plasticizers and cementitious materials as an electron donor.

Table 7 shows values of k3 and bH obtained by this study compared with other values reported in the literature (Henze et al. 2000;Duthy 1993). A typical value for k3, the maximum rate constant (corresponding to μ_H_) in the ASM, is 1.0 to 2.0 d^-1^. This is about ten times larger than the k3 = 0.1 d^-1^ found in this study. However, typical values of bH in the ASM is 0.05 to 0.1 d^-1^ and is about 2.5 to 10% of the maximum rate constant, which is compatible with the relation of bH and k3 (5%) found in this study. The k3 reported for the experiment using ethyl alcohol as an electron donor (Duthy 1993) was also ten times larger than the k3 value found in this study.

**Table 7:** **Comparison of parameters between this study and previously reported values in the literature** (Henze et al. 2000;Duthy 1993)

Source of data	k3	bH	bH/k3	Remarks
	d^-1^	d^-1^	%	
This study	0.1	0.005	5	Obtained values calculated for extract from the mixture of super plasticizer and cementitious materials
Henze et al. (2000)	1.0 - 2.0	0.05 - 0.1	2.5 - 10	Typical values shown as ASM's parameter for organics contained in municipal sewage.
Duthy (1993)	2.4 - 3.1	0.048	1.5 - 2.0	Parameter obtained by one dimensional soil column using ethyl alcohol as organic materials

From the *in-vitro* experiments, the extract of super plasticizers is a more bio-degradable organic material than humic acids, but less bio-degradable than easy-decomposable organic materials such as citric acid. The results shown in Table 7 support such an interpretation. Furthermore, the fact that bH was found to be low suggests that microorganisms originated from actual groundwater have relatively slow cycles of growth and death using hard-biodegradable organics instead of easy-biodegradable organics.

## Conclusions

A series of *in-vitro* and *in-situ* experiments were conducted to investigate the NO_3_^–^ reduction reaction relevant to the geological disposal of NO_3_^–^ containing radioactive wastes. The NO_3_^–^ reduction reaction rate was found to be strongly affected by the degree of degradability of the existing organic materials. Data obtained from the *in-vitro* experiments, using activated sludge or microorganisms originating from an underground environment suggested that de-nitrofication rate using super plasticizer as electron donors were higher than humic acids. In the case of humic acids, the rate was found very low so that organics originated from dead microorganism could act as electron donors. The results were used to develop a NO_3_^–^ reduction reaction model. The model was verified by simulating data obtained from *in-situ* experiments using actual groundwater and microorganisms and provided a good correlation microorganism growth and organics consumption. Although obtained de-nitrification rate parameter for extracts from super plasticizer in the in-situ experiment was about one tenth of those obtained by the experiments using more decomposable organic compounds, nitrated reduction in near field of the repository might occur.
